# Soluble receptors in cancer: mechanisms, clinical significance, and therapeutic strategies

**DOI:** 10.1038/s12276-023-01150-6

**Published:** 2024-01-05

**Authors:** Eun-Ji Park, Chang-Woo Lee

**Affiliations:** 1 Department of Molecular Cell Biology, Sungkyunkwan University School of Medicine, Suwon, Republic of Korea; 2 SKKU Institute for Convergence, Sungkyunkwan University, Suwon, Republic of Korea

**Keywords:** Targeted therapies, Tumour biomarkers

## Abstract

Soluble receptors are soluble forms of receptors found in the extracellular space. They have emerged as pivotal regulators of cellular signaling and disease pathogenesis. This review emphasizes their significance in cancer as diagnostic/prognostic markers and potential therapeutic targets. We provide an overview of the mechanisms by which soluble receptors are generated along with their functions. By exploring their involvement in cancer progression, metastasis, and immune evasion, we highlight the importance of soluble receptors, particularly soluble cytokine receptors and immune checkpoints, in the tumor microenvironment. Although current research has illustrated the emerging clinical relevance of soluble receptors, their therapeutic applications remain underexplored. As the landscape of cancer treatment evolves, understanding and targeting soluble receptors might pave the way for novel strategies for cancer diagnosis, prognosis, and therapy.

## Decoding soluble receptors: the future of cancer prognosis and immune evasion

Soluble receptors, a kind of cellular receptor that exist in a dissolvable form and can boost or interrupt cellular signaling pathways, are the focus of this study. Abnormal amounts of these receptors are associated with the severity of many diseases, including cancer. This research by Eun-Ji Park and Chang-Woo Lee delves into the role of soluble receptors in cancer, specifically soluble cytokine receptors and soluble immune checkpoints. Their study involved a thorough review of existing literature and data on these soluble receptors. They discovered that high levels of soluble receptors are found in the blood of cancer patients, suggesting their potential use as minimally invasive indicators for early cancer detection and prognosis. The authors also suggest that blocking these soluble receptors could enhance the effectiveness of current cancer treatments.

This summary was initially drafted using artificial intelligence, then revised and fact-checked by the author.

## Introduction

Soluble receptors are unique types of cellular receptors that exist in a soluble form. Receptors generally consist of a cytoplasmic domain, a transmembrane domain, and an extracellular domain. Soluble receptors are released into the extracellular space in the form of an extracellular domain lacking a transmembrane domain or bound to extracellular vesicles^1^. By binding to ligands in the extracellular environment, independent of their membrane-bound counterparts, soluble receptors can enhance or disrupt cellular signaling pathways^2^. They can also enter the circulation and elicit local and systemic effects by regulating cellular processes in various physiological conditions^3^. However, abnormal levels of these receptors in the circulation have been associated with disease severity across a range of conditions, including autoimmune diseases, diabetes, infectious diseases, and cancer^3–6^.

In cancer research, soluble receptors have recently generated interest due to their potential as biomarkers^5,7,8^, given their increased levels in the bodily fluids of patients. As biomarkers, they might offer benefits in early detection of cancer, prognosis estimation, and monitoring of treatment response. Beyond their diagnostic value, emerging evidence has demonstrated that soluble receptors are involved in cancer progression, metastasis, and escape from immune surveillance^9–12^. Specifically, soluble forms of cytokine receptors and immune checkpoints have been identified as key modulators in cancer pathogenesis. Although their clinical relevance in cancer has become increasingly apparent, their therapeutic use remains a budding field. Given the limitations of current cancer therapies, targeting soluble receptors is expected to open promising therapeutic avenues.

This review endeavors to dissect the complexities of soluble receptors in cancer. We will elucidate key mechanisms of soluble receptors, from their generation to their roles in cancer pathogenesis, with a particular focus on soluble cytokine receptors and soluble immune checkpoints. Additionally, we will delve into their clinical significance across multiple cancer types, reflecting on current research and existing therapeutic challenges. As our comprehension of soluble receptors evolves, this review highlights their potential as diagnostic/prognostic biomarkers and therapeutic targets in cancer.

## Generation of soluble receptors

**Fig. 1:**
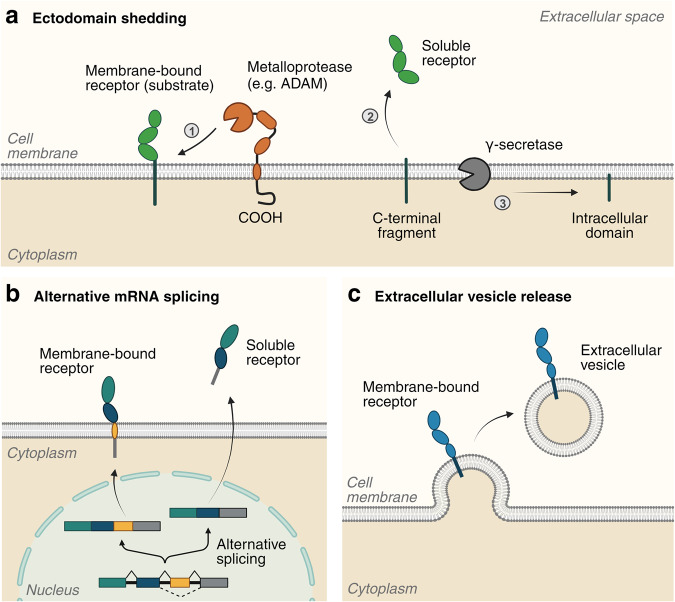
Different mechanisms of soluble receptor generation. **a** Ectodomain shedding of a membrane-bound receptor. The substrate receptor is cleaved by an ADAM protease, resulting in release of a soluble receptor into the extracellular space. The remaining C-terminal fragment is further cleaved by the γ-secretase protease complex to generate an intracellular domain fragment. **b** Alternative splicing of a transcript encoding a receptor, generating either a membrane-bound receptor or a soluble receptor. **c** Release of a membrane-bound receptor in extracellular vesicles. This figure was created with BioRender.com.

Given the importance of soluble receptors in the development of various diseases, a comprehensive understanding of the mechanisms involved in their generation is essential for identifying potential therapeutic targets. Soluble receptors are known to be produced by several distinct molecular mechanisms, including (1) ectodomain shedding, (2) alternative mRNA splicing, and (3) extracellular vesicle release (Fig. 1). In this section, the generation of soluble receptors by each mechanism and clinical implications will be discussed.

### Ectodomain shedding

Ectodomain shedding is a process in which transmembrane proteins exposed on the cell surface or cellular organelles are proteolytically cleaved and released by enzymes, called “sheddases”^13^. The cleaved extracellular domain (ectodomain) of a membrane-bound receptor is released into the extracellular space and transported in a soluble form. Enzymes known as ADAMs (a disintegrin and metalloproteinases), which are the best-characterized sheddases, are central to this process (ectodomain shedding). Within this ADAM family, ADAM10 and ADAM17, which have similar structures, are of particular interest, especially in the context of cancer research^14–16^. They consist of a catalytic metalloproteinase domain that functions in shedding, a disintegrin domain, a cysteine-rich domain, a transmembrane domain, and a C-terminal cytoplasmic domain that is involved in activity regulation. The short C-terminal fragment (CTF) that remains at the plasma membrane as a result of receptor cleavage is further processed by the γ-secretase protease complex to release the intracellular domain (ICD) fragment (Fig. 1a). Although most ICDs are degraded, some are translocated to several cellular compartments such as the nucleus and mitochondria where they are involved in intercellular signaling^17^.

Ectodomain shedding is known as a general mechanism for generating soluble forms of growth factor receptors and many types of cytokine receptors^18^. For instance, cytokine receptors cleaved by sheddases include class I cytokine receptors (e.g., IL-2 receptor, IL-6 receptor), the tumor necrosis factor (TNF) receptor superfamily, and the IL-1 receptor /Toll-like receptor superfamily^3,19,20^. Recent studies have indicated that serum levels of soluble receptors generated by proteolytic cleavage are correlated with disease severity in patients^4,14,21^. To date, considerable research has illuminated the mechanisms and roles of soluble receptors produced through ectodomain shedding. However, the underlying mechanisms governing shedding and soluble receptor generation remain elusive.

### Alternative mRNA splicing

Soluble receptors can also be generated through alternative mRNA splicing, which can remove the exon encoding transmembrane domain of the receptor. When a full-length receptor is expressed, it exists in a form bound to the cell membrane through the transmembrane domain. However, when soluble form of the receptor lacking transmembrane region is expressed, it is secreted from the cell into the extracellular space (Fig. 1b). Recent studies have revealed that many soluble cytokine receptors are generated by alternative splicing as well as ectodomain shedding^19,22–24^. TNF receptor 2 (TNFR2) can undergo alternative splicing to produce a soluble isoform that lacks exons 7 and 8, which encode transmembrane and cytoplasmic domains^25^. This soluble TNFR2 (sTNFR2) can be detected in human serum and its levels are elevated in patients with cancer and inflammatory diseases^26–28^. In addition to cytokine receptors, several inhibitory immune checkpoints have been shown to be released in a soluble form by alternative splicing. A soluble form of PD-1 (programmed death 1) is generated by alternative splicing of exon 3, which encodes the transmembrane domain of the PD-1 gene^29^. Another immune checkpoint CTLA-4 (cytotoxic T lymphocyte antigen 4) is also found in a soluble form lacking the transmembrane domain, encoded by exon 3 of CTLA-4 gene^30^. These soluble immune checkpoints can be detected in human serum and used as diagnostic markers in patients with various cancers^5,9,31^.

### Extracellular vesicle release

Membrane-bound receptors are also released as components of extracellular vesicles such as microvesicles and exosomes. Although the receptor itself is not in a soluble form, it remains bound to the vesicle membrane. It is then released into the extracellular space, where it can still bind to its ligand (Fig. 1c). Some cytokine receptors such as TNF receptors (TNFR1 and TNFR2) and IL-6 receptor (IL-6R) have been detected on extracellular vesicles as full-length proteins^32–34^. These circulating vesicles can affect signaling pathways in other cell types. Additionally, it has been reported that tumor-derived exosomes can carry immunosuppressive or immunostimulatory molecules on their surface to mediate the function of immune cells in the tumor microenvironment^35^. ADAM proteases have also been found in extracellular vesicles such as exosomes^36,37^, suggesting that the ectodomain of receptors on the vesicle membrane might be cleaved and released by ADAM in these vesicles. However, shedding from extracellular vesicles remains largely unexplored.

It is noteworthy that extracellular vesicles such as exosomes can fuse with other cells^38^. This suggests that cells that do not normally express a particular receptor can express that receptor in its full-length upon fusion with such extracellular vesicles. It has been reported that extracellular vesicles containing full-length IL-6R can be fused with distant cells lacking IL-6R, inducing long-term intracellular signaling in target cells^39^. In cancers, microvesicles containing epidermal growth factor receptor variant III (EGFRvIII) are released from glioma cells and transferred to other cells lacking EGFRvIII, leading to a transformed phenotype^40^. Similarly, EGFR-containing exosomes can be transferred from primary gastric cancer cells to liver stromal cells and promote liver metastasis^41^. Therefore, extracellular vesicles with full-length receptors are critical for tumor progression.

## Soluble cytokine receptors in cancer

Cytokines as messengers of the immune system can modulate immune responses by orchestrating cellular functions including cell proliferation, differentiation, and migration^42^. When receptors are bound by their respective cytokines, they initiate a series of intracellular signaling cascades. Numerous studies have reported that their dysregulation is closely associated with the pathogenesis of inflammatory diseases and cancer^42–44^. In the context of cancer, prolonged activated or suppressed signaling of certain cytokines can foster immune evasion in the tumor microenvironment^45,46^. Moreover, some cytokines and their receptors can be produced by tumor cells themselves, creating an autocrine loop that further enhances cell survival and proliferation^47,48^.

Cytokine receptors are found in membrane-bound form and soluble forms. Soluble forms of cytokine receptors can be released into the extracellular environment, which adds another layer of regulation. By binding freely to their respective ligands, they either enhance or reduce cytokine signaling depending on the context, thereby regulating tumor growth and the surrounding microenvironment^10,11,49^. Notably, levels of soluble cytokine receptors have been reported to be higher in serum of patients with various cancers than in that of healthy controls (Table 1). The next section will detail the mechanisms and clinical significance of representative soluble cytokine receptors, including the soluble forms of IL-2 receptor, IL-6 receptor, and TNF receptors.

**Table 1:** Biological functions of soluble cytokine receptors and clinical significance in cancer patients.

Soluble cytokine receptor	Biological function	Cancer types associated with high serum levels of soluble cytokine receptors	Ref.
Soluble IL-2R	1. Binds antagonistically to IL-2 and inhibits proliferation of CD8^+^ T cells. 2. Inhibits the function of effector T cells by enhancing IL-2-induced STAT5 phosphorylation and promoting regulatory T (Treg) cells development.	Colorectal cancer	^120–122^
		Gastric cancer	^96,122–124^
		Esophageal squamous cell carcinoma	^125,126^
		Head and neck cancer	^56,127,128^
		Lymphoma	^57,129^
Soluble IL-6R	Binds to IL-6, forming IL-6/sIL-6R complex and activating cells through gp130 homodimers (trans-signaling)	Multiple myeloma	^130–132^
		Breast cancer	^133–135^
		Lung cancer	^10^
		Leukemia	^136^
Soluble TNFR2	Inhibits the action of TNF-α and induces immune suppression through activation of Treg cells	Colorectal cancer	^137–139^
		Ovarian cancer	^140–142^
		Breast cancer	^134,135^
		Lymphoma	^143–146^
		Lung cancer	^147,148^
Soluble IL-15R	Enhances the activity of IL-15 to induce expansion of CD8^+^ cells and natural killer (NK) cells	Head and neck cancer	^11^
		Leukemia	^149^

### Soluble IL-2R (sIL-2R)

Interleukin-2 (IL-2) is a cytokine critical for T cell proliferation, the generation of effector and memory CD8^+^ T cells, and the cytotoxic activity of natural killer (NK) cells^50^. The IL-2 receptor (IL-2R) comprises three subunits: IL-2Rα (CD25), IL-2β (CD122), and γ-chain (γc, CD132). Of these, the α subunit can be shed by proteases from the cell surface to form soluble IL-2 receptor (sIL-2Rα)^51–53^. In serum, sIL-2Rα can modulate the biological function of IL-2 by either diminishing or enhancing IL-2-mediated effects, depending on the context. It has been reported that sIL-2Rα can compete with membrane-bound IL-2R for IL-2 binding and inhibit IL-2-mediated proliferation of memory phenotype CD8^+^ T cells in vitro^54^. In contrast, sIL-2Rα can facilitate IL-2-mediated STAT5 activation and induce Foxp3 expression in CD4^+^ T cells, which is critical for the generation and maintenance of regulatory T (Treg) cells, suppressing CD8^+^ T cell proliferation^55^.

Previous studies have reported that levels of sIL-2Rα are increased in patients with many cancers, including carcinoma and lymphoma^56–58^. Elevated sIL-2Rα level is correlated with high grade tumors and poor overall survival^56,59^, suggesting that it can be used as a non-invasive marker for the diagnosis and prognosis of cancer. Given that sIL-2R regulates immune responses, understanding its role in the tumor microenvironment can pave the way for novel anti-tumor therapies. Therefore, its clinical significance as a biomarker and a potential therapeutic target for cancer warrants further investigation.

### Soluble IL-6R (sIL-6R)

The interleukin-6 (IL-6) signaling pathway is critical for various physiological processes, including inflammation, hematopoiesis, metabolism, and cancer^60^. The IL-6 receptor (IL-6R) exists in two forms: membrane-bound IL-6R and its soluble counterpart. In IL-6 classic signaling, IL-6 binds to membrane-bound IL-6R, inducing homodimerization of signal transducer protein gp130 (CD130) and activation of intracellular signaling cascades^61,62^. Soluble IL-6R (sIL-6R) can be generated by ectodomain shedding, alternative splicing, and release on extracellular vesicles^19^. sIL-6R retains its ability to bind to IL-6, forming the IL-6/sIL-6R complex. This complex then associate with membrane-bound gp130 homodimers, leading to intracellular signaling. This process is called IL-6 trans-signaling^63^. Notably, while IL-6 classic signaling through membrane-bound IL-6R is restricted to specific cell types such as hepatocytes and some lymphoid cells, IL-6 trans-signaling via sIL-6R can occur in all cells. It has been reported that gp130 is ubiquitously expressed in almost all cells except granulocytes^19^.

It is known that the IL-6-induced JAK/STAT3 signaling pathway drives the proliferation and survival of tumor cells^62^. Indeed, IL-6 trans-signaling has been reported to promote the development of pancreatic cancer and KRAS-driven lung adenocarcinoma^10,64^. In colitis-associated cancer (CAC), IL-6 and sIL-6R are produced by lamina propria myeloid cells. They stimulate the proliferation of premalignant intestinal epithelial cells, affecting early tumor formation^65,66^. During the late stages of CAC development, tumor-derived sIL-6R rather than membrane-bound IL-6R induces STAT3 activation and accelerates tumor growth^67,68^. In addition to signal transduction in tumor cells, IL-6 trans-signaling in immune cells affects tumor progression. It has been revealed that IL-6 trans-signaling via sIL-6R derived by myeloid cells attenuates CD4^+^ T helper type 1 (Th1) cell differentiation in tumor-bearing mice, leading to defective anti-tumor responses^69^. Moreover, IL-6 trans-signaling promotes immunosuppressive function of myeloid-derived suppressor cells (MDSCs) in breast cancer^70^. Given the role and significance of IL-6/sIL-6R trans-signaling in tumor progression, targeting this trans-signaling has therapeutic potential in many types of cancer.

### Soluble TNFR (sTNFR)

Tumor necrosis factor (TNF) is a multifunctional cytokine that plays a role in homeostasis and disease pathogenesis^71^. TNF binds to two distinct receptors: TNF receptor 1 (TNFR1) and TNF receptor 2 (TNFR2). TNFR1 and TNFR2 have similar extracellular structures and are activated by both soluble and transmembrane TNF^26^. TNFR1 has an intracellular death domain and induces inflammation and tissue degeneration as well as programmed cell death. In contrast, TNFR2 lacks a death domain and mediates primarily homeostatic effects, including cell survival, proliferation, and tissue regeneration^71^. Both TNFR1 and TNFR2 can exist in soluble and membrane-bound forms. Soluble TNFRs are generated by proteolytic cleavage, synthesis via alternative mRNA splicing, or release in extracellular vesicles^3^. TNFRs are cleaved by ADAM17, also known as TACE (TNF-α converting enzyme)^72^. It has been shown that levels of soluble TNFR1 (sTNFR1) and soluble TNFR2 (sTNFR2) are increased in several diseases, including type 1 and type 2 diabetes with chronic kidney diseases^73,74^.

Previous studies have shown that the TNFR2-expressing Treg subset has a highly immunosuppressive function^75,76^. Additionally, TNFR2 has been reported to be expressed on CD8^+^ regulatory T cells (CD8^+^ Tregs) and CD8^+^ effector T cells, thus coordinating immune responses^77^. It is noteworthy that TNFR2 is increased in tumor-infiltrating Treg cells from human solid tumors^78^. In murine lung cancer and melanoma models, tumor growth in TNFR2-deficient mice was significantly decreased compared to that in wild-type mice^79^. Levels of sTNFR2 in serum/plasma samples of several cancer patients are elevated, and such elevation has been found to be correlated with cancer development and poor overall survival^80^. These findings suggest that circulating sTNFR2 plays a pivotal role in the tumor microenvironment and can be used as a biomarker for cancer diagnosis and prognosis as well as cancer therapy.

## Soluble immune checkpoints in cancer

Immune checkpoints are paired receptor-ligand molecules that fine-tune the immune system to maintain immune homeostasis. Recently, immune checkpoints have gained attention in cancer immunotherapy, due to their exploitation by tumor cells rather than their protective role^81,82^. Overall, discovery of their roles in tumor immune evasion has paved the way for immune checkpoint blockade as a revolutionary therapeutic approach. In recent decades, antibodies targeting immune checkpoints, such as CTLA-4 and PD-1, have been actively developed and studied for cancer treatment^82^. However, clinical trials of immune checkpoint blockade to date have revealed limitations, as the percentage of patients who respond to such treatment is still low^83^. For example, although combination therapy with anti-PD-1 antibody nivolumab and anti-CTLA-4 antibody ipilimumab has been demonstrated to have therapeutic effects on overall survival outcomes in patients with advanced melanoma^84,85^, only a few patients can benefit from this treatment. Due to such limitations in the development of immunotherapies for cancer, an in-depth understanding of the mechanism of immune checkpoints has become necessary.

In addition to being expressed on cell membranes, immune checkpoints can be found in soluble form. Several studies have identified the source of specific soluble immune checkpoints. For example, soluble form of programmed cell death ligand 1 (sPD-L1) is reported to be produced by tumor cells or activated mature dendritic cells^86,87^. sPD-L1 can be generated via ectodomain shedding and binds to PD-1 to inhibit T cell responses^87,88^. Moreover, it has been reported that soluble CTLA-4 (sCTLA-4) is produced by Treg cells through alternative mRNA splicing^89^, and the spliced variant has also been detected in monocytes and immature dendritic cells^90^. Although the major sources of several soluble immune checkpoints have been identified in vitro, the molecular mechanisms responsible for the generation and physiological function of soluble immune checkpoints in vivo still require further investigation.

Recent studies have shown that soluble forms of immune checkpoints can be detected in human serum or plasma and that elevated levels are associated with many cancer types^7,9,91,92^. Levels of these soluble immune checkpoints are not only simply increased in cancer patients but also correlated with the disease severity and prognosis of patients (Table 2). Moreover, high serum levels of soluble immune checkpoints are associated with resistance to several targeted cancer therapies in cancer patients^93–95^. These findings suggest the potential of using circulating immune checkpoints as biomarkers for the diagnosis and prognosis of various cancers.

**Table 2:** Clinical significance of soluble immune checkpoints in cancer.

Cancer type	Soluble immune checkpoint	Study outcome in cancer patients	Ref.
Clear cell renal cell carcinoma (ccRCC)	sPD-L1 ↑	Elevated levels are associated with aggressive renal cell carcinoma	^86^
	sTIM3 ↑	Increased levels are associated with advanced disease (stage III)	^9^
	sLAG3 ↑		
Non-small cell lung cancer (NSCLC)	sLAG3 ↑	Significantly elevated in early-stage patients (stages I and II)	^150^
	sTIM-3 ↑ sCD137 ↑ sCD27 ↑	Significantly elevated in advanced patients (stages III and IV)	
	sB7‐H3 ↑	Elevated levels are associated with poor prognosis (higher tumor stage, metastasis)	^151^
	sCD40 ↑	Elevated levels are associated with advanced diseases and poor prognosis	^152^
	sPD-L1 ↑	Elevated levels are associated with poor prognosis	^153^
Hepatocellular carcinoma (HCC)	sPD-1 ↑	Plasma levels are associated with HCC risk for men	^154^
	sPD-L1 ↑	High levels are associated with the stages of HCC and cirrhosis and mortality	^155^
Leukemia	sCTLA-4 ↑	Significantly elevated in acute lymphoblastic leukemia (ALL) pediatric patients and the levels are correlated with negative prognostic marker CD1d	^156^
	sCD80 ↑	Elevated levels are associated with poor prognostic markers in chronic lymphocytic leukemia (CLL)	^157^
Lymphoma	sPD-L1 ↑	High levels are associated with poor overall survival in aggressive diffuse large B-cell lymphoma (DLBCL)	^158^
		High levels are correlated with advanced stage of Hodgkin lymphoma (HL) and poor prognosis	^159^
		High levels are associated with poor prognosis in natural killer/T-cell lymphoma (NKTCL)	^160^

## Targeting soluble receptors for cancer therapy

### Therapeutics that directly target soluble receptors

**Fig. 2:**
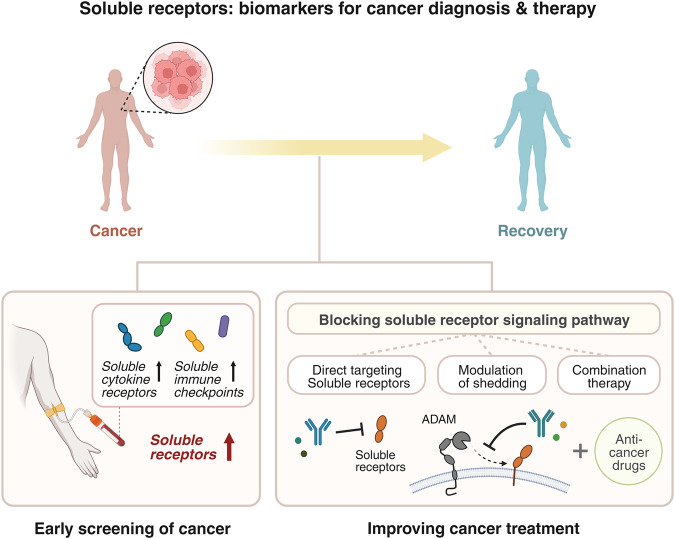
Soluble receptors as biomarkers for cancer diagnosis and therapy. Targeting soluble receptors has benefits in cancer diagnosis, prognosis, and treatment. Considering that high levels of soluble receptors are detected in the bodily fluids of cancer patients and that such high levels are associated with disease severity, soluble receptors have the potential to be used as minimally invasive biomarkers for early detection and prognosis of cancer. Additionally, blocking soluble receptors through various therapeutic strategies can potentially improve the efficacy of current cancer treatment. This figure was created with BioRender.com.

Soluble receptors not only have the potential to be used as biomarkers for cancer diagnosis and prognosis, but can also be used as therapeutic targets in cancer treatment (Fig. 2). Direct targeting of soluble receptors or their pathways might be an effective therapeutic strategy to enhance anti-tumor responses. Although additional research is still needed, the clinical significance of increased soluble receptor levels in patients with various types of cancer provides sufficient evidence to support the development of novel cancer treatments by targeting these soluble receptors. Circulating levels of sIL-2Rα, one of the soluble cytokine receptors, have been shown to be correlated with progression of several types of cancer in previous studies^96–98^, with potential for use as a diagnostic and prognostic marker for cancer. However, considering that it is highly correlated not only with cancer but also with other diseases such as inflammatory diseases^3^, it can be used as an indicator to confirm the activation of T cells that produce sIL-2Rα in pathogenic conditions rather than simply as an indicator of cancer^49^. Therefore, when directly targeting soluble receptors as a cancer treatment strategy, it is important to conduct a precise analysis according to the characteristics of each cancer type and understand the molecular mechanism of each soluble receptor. To date, small-molecule compounds or monoclonal antibodies that directly target several soluble receptors have been developed, and research into the mechanism of how these substances affect the tumor microenvironment and their efficacy in both preclinical and clinical trials is ongoing.

One of the therapeutic strategies being actively investigated is blocking IL-6 trans-signaling^99,100^. Some humanized monoclonal antibodies targeting IL-6R, such as tocilizumab and sarilumab, have been developed to inhibit IL-6 signaling. They are currently approved for treating arthritis or are in clinical trials for other diseases^101–103^. However, the problem with these antibodies is that they cannot distinguish between membrane-bound and soluble forms of IL-6R, inhibiting IL-6 classic signaling and trans-signaling at the same time. An alternative IL-6 trans-signaling inhibitor that can be considered is a soluble gp130–Fc fusion protein (sgp130Fc, also known as olamkicept), which is a fusion protein of the extracellular portion of gp130 and the Fc region of a human IgG1 antibody^104^. IL-6 classic signaling maintains local homeostasis even under normal healthy conditions; under inflammatory conditions, however, local IL-6 classic signaling and trans-signaling as well as systemic trans-signaling are induced by high levels of IL-6 and sIL-6R in the blood^100^. Therefore, sgp130Fc, which selectively inhibits only trans-signaling without affecting IL-6 classic signaling, has high potential as a therapeutic agent for various diseases. As mentioned above, IL-6 trans-signaling in various cancer types promotes tumor progression by suppressing anti-tumor responses of immune cells as well as increasing cell growth through signal transduction in tumor cells themselves. Surprisingly, blocking IL-6 trans-signaling with sgp130Fc has therapeutic effects on reducing tumor progression in murine cancer models, including murine colitis-associated cancer (CAC)^65,66^, lung adenocarcinoma^10^, and hepatocellular carcinoma (HCC) models^105,106^. However, few studies on blockade of IL-6 trans-signaling in cancer patients have been reported, despite its therapeutic effects in numerous preclinical cancer models. Therefore, additional clinical studies are needed before it can be used as a clinical tool for cancer treatment.

Another strategy to directly target soluble receptors for cancer treatment is to inhibit sTNFR. TNFR2-expressing Tregs are increased in the tumor microenvironment and have a high suppressive capacity in various cancers, including ovarian cancer, acute myeloid leukemia, and lung cancer^107–109^. It has been revealed that newly identified TNFR2 antagonistic monoclonal antibodies (TNFR2 antagonists) inhibit soluble TNFR2 secretion and Treg proliferation in vitro^110^. Of note, a TNFR2 antagonist has a greater effect on suppressing Tregs from the ascites fluid of ovarian cancer patients than Tregs from the peripheral blood of healthy donors^110^, suggesting the specificity of TNFR2 antagonists for the tumor microenvironment. Specifically, inhibiting the activity of tumor-residing Tregs through TNFR2 antagonism can increase proliferation of effector T cells in the tumor microenvironment and suppress tumor growth. Thus, it can be considered an engaging cancer therapy.

### Modulation of ectodomain shedding

Inhibiting enzymes responsible for shedding of membrane-bound receptors can reduce levels of soluble receptors, which might also be a therapeutic strategy for cancer. Recent studies have shown that expression levels of ADAMs are increased in multiple cancer types^111,112^. Preclinical studies have reported that small-molecule compounds or monoclonal antibodies for modulating ADAMs inhibit migration, invasion, and growth of tumor cells^112^. For instance, treatment with Aderbasib (INCB7839), a small-molecule inhibitor of ADAM10/ADAM17, was reported to prevent growth of HER2^+^ human breast cancer in a mouse xenograft model^113^. INCB7839 has also been tested in clinical trials, with promising results in phase I/II trials of Trastuzumab-based HER2^+^ breast cancer therapy by inhibiting HER2 shedding (NCT01254136), and evaluated in phase I trials for recurrent or progressive high-grade gliomas (NCT04295759).

Targeting catalytic domains (metalloprotease domains) of the ADAM protease has so far failed due to highly conserved active sites among ADAM enzymes, resulting in unfavorable toxic effects. Interestingly, recent studies have revealed that non-catalytic domains of ADAM10 and ADAM17, specifically a disintegrin domain and a cysteine-rich domain, can provide substrate specificity^114^. Through additional research, it is expected that inhibitors with increased specificity for other ADAM families with different structures will be developed, which will provide a way to overcome the limitations in ADAM inhibitor development. There are several ongoing clinical trials targeting specific ADAM proteases for cancer. The ADAM9-targeting antibody-drug conjugate IMGC936 has been tested in phase I/II trials for advanced solid tumors, such as non-squamous non-small cell lung cancer, triple-negative breast cancer, and colorectal cancer (NCT04622774). Therefore, targeting specific ADAM proteases may be a promising therapeutic strategy for cancer.

### Enhancers of existing therapies: combination therapy

Understanding the interplay between soluble receptors and established therapeutic strategies can lead to more effective treatments. Despite the clinical success of current cancer immunotherapies, such as immune checkpoint blockade, a significant proportion of cancer patients still do not respond to treatment or are resistant to inhibitor treatment^115^. Of note, serum levels of soluble immune checkpoints are correlated with resistance to immunotherapy. In patients with advanced melanoma, anti-PD-1 antibody (pembrolizumab) monotherapy has been reported to increase serum levels of lymphocyte-activation gene 3 (sLAG3) in a disease progression group compared to a control group^95^. Additionally, serum PD-1 levels are increased in melanoma patients with disease progression, following combination treatment with anti-PD-1 antibody (nivolumab) plus anti-CTLA-4 antibody (ipilimumab)^95^. These recent findings suggest that targeting soluble immune checkpoints might be beneficial for immunotherapy-resistant cancer patients. Therefore, combination therapy with existing therapeutic strategies and inhibition of soluble receptors might be a solution to overcome the limitations of current treatments.

### Challenges and future directions

Therapeutic strategies for cancer by inhibiting IL-6 trans-signaling should consider the effects of other IL-6 family members. The IL-6 family consists of IL-6, IL-11, and IL-27, all of which transduce signals using the gp130 receptor^20^. Of note, the IL-11 receptor (IL-11R) can also be detected in a soluble form. Levels of soluble IL-11R have been reported to be elevated in patients with gastric cancer^116^, suggesting possible effects of IL-11 trans-signaling in cancer progression in vivo. To reduce the potential of side effects, second-generation and third-generation variants have been developed from the previously developed sgp130Fc^100^. Indeed, the selectivity of inhibitors for IL-6 trans-signaling has gradually increased from the first-generation sgp130Fc to the second-generation variant sgp130^FLYR^Fc and the third-generation variant cs130^Fly^, but the effect on IL-11 trans-signaling has gradually decreased^117,118^. Therefore, increasing the selectivity of inhibitors targeting specific soluble receptor signaling will be of great help in reducing unwanted side effects in clinical studies.

In addition, several sheddase inhibitors are currently being developed for cancer treatment. While the specificity between ADAM family members has been addressed to some extent in the development of ADAM-targeted inhibitors, the fact that each ADAM can cleave a variety of substrates, including multiple cytokines, growth factors, and other membrane-bound receptors, may lead to detrimental side effects in clinical studies^18^. A recent study revealed that MEDI3622, a specific ADAM17 inhibitory antibody, has the potential to inhibit not only the HER pathway but also the EGFR pathway^119^. The researchers used combination therapy with MEDI3622 and the EGFR inhibitor cetuximab and found synergic effects resulting in complete tumor regression in an OE21 esophageal xenograft model^119^. Therefore, in future studies of sheddase inhibitors, it is necessary to first analyze expression of multiple substrates in each patient with a specific cancer type.

## Conclusions

Soluble receptors have evolved as crucial players in cancer research. Their generation through various mechanisms, such as ectodomain shedding, alternative mRNA splicing, and extracellular vesicle release, underscores multifaceted ways in which they regulate cellular signaling pathways. Focusing on the roles of soluble cytokine receptors and soluble immune checkpoints, this review highlights the indispensable role of soluble receptors in cancer progression, metastasis, and immune evasion. Soluble receptors are detected at high levels in the blood of patients with various cancers. Given that soluble receptors are present in bodily fluids, they might provide a minimally invasive method for early diagnosis and prognosis of cancer. In addition to early cancer detection, directly targeting soluble receptors using small-molecule compounds or monoclonal antibodies could be considered as cancer treatment strategies. The therapeutic potential of targeting these soluble receptors offers promising avenues for cancer treatment, and strategically combining therapies may enhance the efficacy of current strategies. Nevertheless, in the case of soluble immune checkpoints, how direct regulation of soluble immune checkpoints affects the tumor microenvironment has not yet been elucidated. Hence, it is evident that more studies are needed, especially in harnessing soluble receptors for cancer treatment. Future endeavors in this area should focus on improving therapeutic strategies, addressing identified challenges, and understanding the long-term implications of targeting soluble receptors in cancer.
